# Phenylalkyl Glycosides from the Flowers of *Brugmansia arborea* L. and Their Radical Scavenging Effect and Protective Effect on Pancreatic Islets Damaged by Alloxan in Zebrafish (*Danio rerio*) Larvae

**DOI:** 10.3390/plants12244075

**Published:** 2023-12-05

**Authors:** Hyoung-Geun Kim, Youn Hee Nam, Tong Ho Kang, Nam-In Baek, Min-Ho Lee, Dae Young Lee

**Affiliations:** 1 Graduate School of Biotechnology and Department of Oriental Medicinal Biotechnology, Kyung Hee University, Yongin 17104, Republic of Korea; zwang05@khu.ac.kr (H.-G.K.); panjae@khu.ac.kr (T.H.K.);; 2 Department of Food Technology and Services, Eulji University, Seongnam 13135, Republic of Korea; 3 Department of Herbal Crop Research, National Institute of Horticultural and Herbal Science, RDA, Eumseong 27709, Republic of Korea

**Keywords:** antioxidant, *Brugmansia arborea*, brugmansioside C, phenylalkyl glycoside, zebrafish

## Abstract

The study aimed to investigate the antioxidant and antidiabetic activity of *Brugmansia arborea* L. flower extracts, solvent fractions, and isolated compounds. *B. arborea* L flowers were extracted with aqueous methanol, and concentrated extract was successively partitioned into EtOAc, *n*-BuOH, and H_2_O fractions. Repeated silica gel and octadecyl silica gel column chromatographies for EtOAc and *n*-BuOH fractions led to the isolation of a new phenylalkyl glycoside (**6**), along with five known ones. Several spectroscopic data led to the structure determination of one new phenylalky glycoside as brugmansioside C (named) (**6**) and five known ones as benzyl-*O*-*β*-D-glucopyranoside (**1**), benzyl-*O*-*β*-D-glucosyl-(1→6)-*β*-D-glucopyranoside (**2**), 2-phenylethyl-*O*-*β*-D-glucopyranoside (**3**), 2-phenylethyl-*O*-*β*-D-glucosyl-(1→6)-*β*-D-glucopyranoside (**4**), and 3-phenylpropyl-*O*-*β*-D-glucopyranoside (**5**). The five known ones (**1**–**5**) were isolated from *B. arborea* flowers for the first time in this study. The extract, solvent fractions, and all isolated compounds showed radical scavenging activities using ABTS radical, and EtOAc fraction showed the highest scavenging capacity, whereas compounds 2, 4, and 6 did not display the capacity to use the DPPH radical. The extract, solvent fractions, and all isolated compounds showed a protective effect on pancreatic islets damaged by alloxan treatment in zebrafish larvae. The pancreatic islet size treated with EtOAc, *n*-BuOH fractions, and all compounds significantly increased by 64.0%, 69.4%, 82.0%, 89.8%, 80.0%, 97.8%, 103.1%, and 99.6%, respectively, compared to the alloxan-induced group. These results indicate that *B. arborea* flowers and their isolated compounds are useful as potential antioxidant and antidiabetic agents.

## 1. Introduction

Diabetes mellitus (DM), a metabolic disease, is characterized by an abnormally high level of serum glucose caused by insulin deficiency or resistance. DM is classified as type 1 and type 2 according to etiology and characteristics [1,2,3]. Type 1 DM (insulin-dependent) is closely related to autoimmunity. Patients with type 1 DM show insulin deficiency due to the destruction of pancreatic *β*-cells. Type 2 DM (non-insulin-dependent diabetes mellitus) is related to hyperglycemia and insulin resistance [4]. DM can lead to serious microvascular complications that result in retinopathy and nephropathy [5]. In the search for antidiabetic drugs, the zebrafish model has been widely used because of its physiological and genetic similarities to mammalian systems [6] as well as its size, handy conservation in laboratories, abundant offspring, transparent embryos, and obedience to genetic and chemical screens [7]. Zebrafish can be treated with alloxan to damage the pancreatic islets, resulting in variations in islet size and glucose absorption.

*Brugmansia arborea* L. (Solanaceae), an evergreen shrub distributed in America, Africa, Australia, and Asia [8], is also called ‘angel’s trumpet’ because the flowers resemble the long trumpet envisioned of angels. The plant reaches up to 3–11 m in height. The ovate leaves are coarsely toothed, and the flowers are strongly fragrant. *B. arborea* has been commonly used for ornamental purposes [8]. In ancient South American Indian culture, *B. arborea* was used as a hallucinogenic drug for rituals, medical supplies, and poison. In particular, *B. arborea* was force-fed to the wives, children, and slaves of Native American rulers who died so that all could be buried together. This plant has also been used for its analgesic, antirheumatic, vulnerary, decongestant, and antispasmodic activities [8,9,10]. Anti-cholinergic activity of *B. arborea* has also been reported [11,12], with the principal compounds revealed to be tropane alkaloids. Despite the several practical uses and reported pharmacological activities of *B. arborea*, the constituents of *B. arborea* flowers are not well described. Accordingly, authors carried out a study to identify the active principals of *B. arborea* flowers.

We isolated and identified one new phenolic glycoside and five known ones. And the extract, solvent fractions, and identified phenolic glycosides were evaluated for the radical scavenging activities using the DPPH and ABTS radicals and the protective effect on pancreatic islets damaged by alloxan treatment in zebrafish larvae. The description of the purification, structure determination, and antioxidant and antidiabetic potential of phenolic glycosides was included in this paper. Since the extract, fractions, and isolated compounds found in this study have diverse variations in their structural characteristics and potent antioxidant and antidiabetic activities, their successive study will lead to the development of safe and effective functional materials against metabolic disease.

## 2. Results and Discussion

### 2.1. Chemical Structure Elucidation

The flowers of *B. arborea* were extracted in aqueous methanol (MeOH) (80%), and the concentrated extracts were partitioned into EtOAc, *n*-BuOH, and water fractions, successively. Among the fractions, both organic fractions yielded one new phenylbutyl glycoside along with five phenylalkyl glycosides.

**Figure 1:**
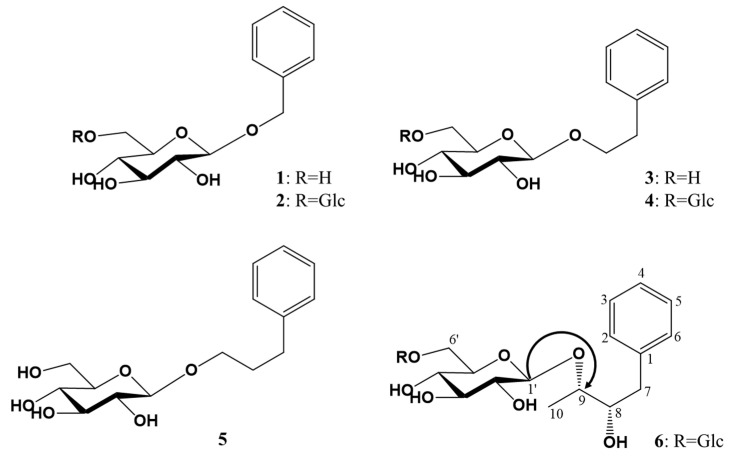
Chemical structures of phenylalkyl glucosides **1**–**6** isolated from the flowers of *Brugmansia arborea*. Glc: *β*-D-glucopyranosyl; the gHMBC key correlations are represented by single-headed arrows from H to C.

The comparison of NMR and MS data with reported values led to the identification of known compounds benzyl *O*-*β*-D-glucopyranoside (**1**) [13], benzyl *O*-*β*-D-glucopyranosyl-(1→6)-*β*-D-glucopyranoside (**2**) [14], 2-phenylethyl *O*-*β*-D-glucopyranoside (**3**) [15], 2-phenylethyl *O*-*β*-D-glucopyranosyl-(1→6)-*β*-D-glucopyranoside (**4**) [14], and 3-phenylpropyl *O*-*β*-D-glucopyranoside (**5**) [16] (Figure 1).

Compound **6** was obtained as a white amorphous powder and became black on the TLC plate after spraying with 10% H_2_SO_4_ and then heating. FT−IR data exhibited the absorption (cm^−1^) of hydroxy (3352) and aromatic C=C (1651, 1528). The molecular weight and molecular formula were, respectively, determined to be 490 and C_22_H_34_O_12_ from the molecular ion peak [M + Na]^+^ *m/z* 513.1943 in the HRFABMS spectrum (calcd. for C_22_H_34_O_12_Na, 513.1948). In the ^1^H-NMR spectrum, the proton signals of five olefin methines δ_H_ 7.26 (m, 1H, H-4), δ_H_ 7.32 (dd, *J* = 8.4, 8.4 Hz, 2H, H-3, 5), δ_H_ 7.41 (br. d, *J* = 8.4 Hz, 2H, H-2, 6) owing to a phenyl moiety, two oxy-methines δ_H_ 3.42 (m, 1H, H-8), δ_H_ 3.94 (m, 1H, H-9), one methylene δ_H_ 2.76 and 2.93 (both dd, both 1H, *J* = 11.0, 3.0 Hz, H-7a; *J* = 11.0, 5.0 Hz, H-7b), and one methyl δ_H_ 1.20 (d, *J* = 5.7 Hz, 3H, H-10) were observed as the signals of an aglycon moiety. The proton signals of two hemiacetal δ_H_ 4.30 (d, *J* = 7.8 Hz, 1H, H-1″), δ_H_ 4.17 (d, *J* = 7.7 Hz, 1H, H-1′), eight oxy-methines δ_H_ 3.17 (overlapped, 2H, H-2′, 2″), δ_H_ 3.31 (overlapped, 2H, H-5′, 5″), δ_H_ 3.35 (overlapped, 2H, H-3′, 3″), δ_H_ 3.57 (dd, *J* = 7.7, 7.7 Hz, 1H, H-4″), δ_H_ 3.72 (overlapped, 1H, H-4′), and two oxy-methylenes δ_H_ 3.76 and 4.05 (both dd, both 1H, *J* = 11.6, 5.2 Hz, H-6′a; *J* = 11.6, 2.0 Hz, H-6′b), δ_H_ 3.65 and 3.86 (both 1H, br. d, *J* = 11.6 Hz, H-6″a; dd, *J* = 11.6, 5.0 Hz, H-6″b) due to two hexose moieties were detected. The above-mentioned ^1^H-NMR spectrum indicated that compound **6** was expected to be a phenylbutyl diglycoside. The ^13^C-NMR spectrum showed 22 carbon signals, confirming compound **6** to be composed of a phenylbutandiol and two hexoses. One olefin quaternary carbon signal (δ_C_ 140.2), three aromatic methine carbon signals (δ_C_ 127.2, 129.4, 130.0), two oxygenated methine carbon signals (δ_C_ 76.6, 88.2), one methylene carbon signal (δ_C_ 39.3), and one methyl carbon signal (δ_C_ 17.4) were observed as the signals of an aglycon, which was identified to be a phenylbutandiol. The chemical shift of the two sugar moieties, including two hemiacetal carbon signals (δ_C_ 104.5, 104.8), eight oxygenated methine carbon signals (δ_C_ 71.6, 71.9, 75.1, 75.2, 77.8, 78.0), and two oxygenated methylene carbon signals (δ_C_ 62.8, 69.9), revealed the sugar to be a *β*-glucopyranosyl-(1→6)-*β*-glucopyranose, and the coupling constant of the anomer proton signal (*J* = 8.0 Hz and *J* = 8.0 Hz) confirmed the two anomer hydroxyls to have *β*-configurations. In the gHMBC spectrum, the two anomer proton signals [δ_H_ 4.30 (H-1′′) and 4.17 (H-1′)] showed a cross-peak with the one oxygenated methine carbon signal [δ_C_ 88.2 (C-9)] and the one oxygenated methylene carbon signal [δ_C_ 69.9 (C-6′)], respectively, suggesting the *β*-D-glucopyranose to be at the C-9 and C-6′ positions. The oxygenated methine and oxygenated methylene carbon signals (C-9 and C-6′) were down-shifted owing to the glycosidation effect from their usual detection at δ_C_ 71.4 and δ_C_ 62.3, respectively, in *β*-D-glucopyranose [17]. The absolute configuration of C-8 and C-9 was proposed as *S* and *S*, respectively, by comparing the optical rotation value (${\left[\alpha \right]}_{D}^{24}$ −23.0°) with that of (2*S*,3*S*)-1-phenyl-2,3-dihydroxybuthyl-3-*O*-*β*-D-glucopyranoside (${\left[\alpha \right]}_{D}^{27}$ −21.9°) [18]. Taken together, these observations identified compound **6** as (2*S*,3*S*)-1-phenyl-2,3-dihydroxybuthyl-*O*-*β*-D-glucopyranosyl-(1→6)-*β*-D-glucopyranoside (Figure 1), which was revealed to be a new compound and named brugmansioside C.

### 2.2. Radical Scavenging Capacity

The antioxidant capacities of extract, solvent fractions, and phenylalkyl glycosides **1**–**6** of *B. arborea* flowers by the ABTS and DPPH assays are shown in Table 1. The experimental information with a description of the measurements of radical scavenging assay was described in a previous study [17]. EtOAc fraction (BAFE) showed the highest antioxidant capacities in both ABTS and DPPH assays. It was thought that the ethyl acetate fraction mainly contained compounds that contribute more to antioxidant capacities. The isolated compounds **1**–**6** showed ABTS radical scavenging capacities in order **1** > **3** > **2** ≥ **5** > **4** > **6**. The DPPH radical scavenging activity was similar to ABTS radical scavenging activity (Table 1). BAFE showed the highest capacity, whereas compounds **2**, **4**, and **6** did not show DPPH scavenging capacity. Because the DPPH assay (80% methanol) measures the radical scavenging ability in the non-polar solvent system, compounds **2**, **4**, and **6,** including two sugars in the structure, showed lower DPPH radical scavenging activity than ABTS [19,20]. Monoglycoside compounds **1** and **3** exhibited higher activity than the diglycoside compounds **2** and **4,** respectively.

### 2.3. Protective Effects on Pancreatic Islets in Zebrafish Treated by Alloxan

**Figure 2:**
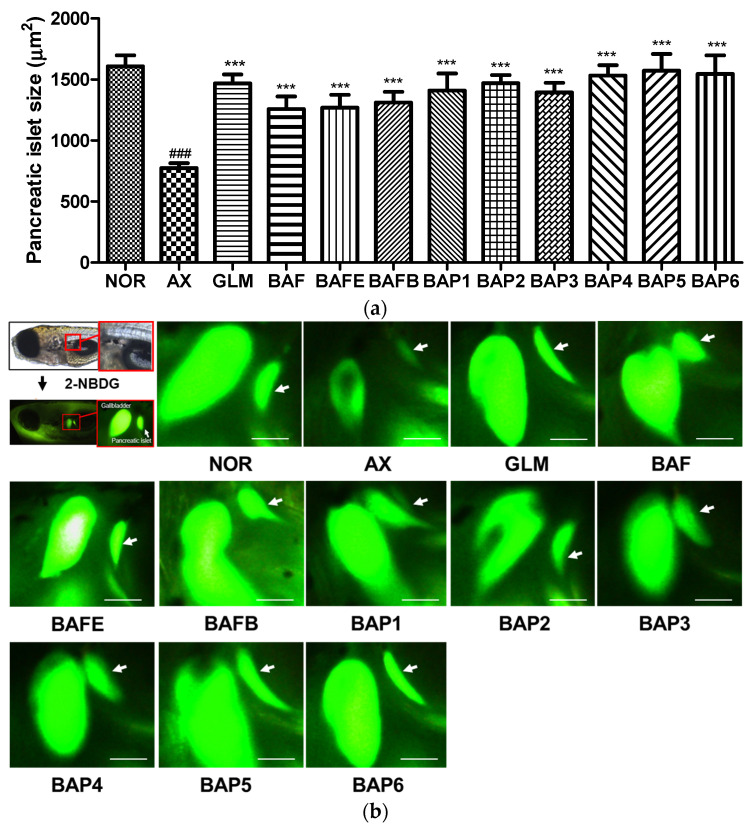
Protective effect of EtOAc and *n*-BuOH fractions and compounds **1**–**6** from *Brugmansia arborea* flowers on alloxan-induced pancreatic islets in zebrafish. (**a**) Size of the pancreatic islets. (**b**) Pancreatic islet image: NOR: normal group, AX: alloxan group, GLM: glimepiride+AX group, BAF: Extract+AX, BAFE: EtOAc fraction+AX, BAFB: *n*-BuOH fraction+AX, BAP1-6: compound **1**–**6**+AX. (^###^ *p* < 0.001; compared to the normal group), (*** *p* < 0.001 compared to the alloxan-treated group). Scale bar = 100 μm.

Extracts, solvent fractions (EtOAc, *n*-BuOH), and isolated phenylalkyl glucosides **1**–**6** from *B. arborea* flowers were evaluated for protective activity against the pancreatic islets of zebrafish larvae damaged by alloxan. The larvae treated with alloxan were used to model type 1 diabetes due to their physiological similarities to mammals [21,22]. Alloxan is a diabetogenic chemical that has been reported to decrease *β*-cell mass in pancreatic islets [23]. To assess pancreatic islets treated with alloxan, the size changes of the pancreatic islets and fluorescence intensities of the NBDG-stained pancreatic islets under a fluorescence microscope were analyzed. When the zebrafish larvae were exposed to alloxan, pancreatic islet size decreased significantly by 51.8% (*p* = 0.0003) compared to the normal group (Figure 2a). Zebrafish larvae treated with glimepiride, a positive control, showed a pancreatic islet increase of 89.5% (*p* = 0.0047) compared to the alloxan group. The pancreatic islet sizes in the groups treated with EtOAc (BAFE) and *n*-BuOH (BAFB) fractions significantly increased up to 64.0 and 69.4% (*p* 0.0091 and 0.0065) compared with alloxan treatment (Figure 2). All of the phenylalkyl glucosides from *B. arborea* flowers also resulted in increases in pancreatic islet size. Compounds **1**–**6** (BAP **1**–**6**) increased the injured pancreatic islets up to 82.0, 89.8, 80.0, 97.8, 103.1, and 99.6% (*p* 0.0011, 0.0037, 0.0012, 0.0002, 0.0011, 0.0011, and 0.0011), respectively, compared with alloxan treatment (Figure 2). All phenylalkyl glucosides isolated from *B. arborea* flowers in this study increased the sizes of pancreatic islets damaged by alloxan treatment in zebrafish larvae with high levels of significance. In particular, phenylpropyl glucoside **5** and the new phenylbutyl glucoside **6** displayed recovery effects greater than glimepiride. These diverse rates also showed structure–activity relationships. Monoglycoside compounds **1** and **3** exhibited lower activity than diglycoside compounds **2** and **4,** respectively. And, compared to compounds **1**, **3**, and **5**, the propyl group attached to phenyl was more effective than the methyl and ethyl groups.

### 2.4. Action of Diazoxide (DZ) on Alloxan-Induced Pancreatic Islets in Zebrafish

**Figure 3:**
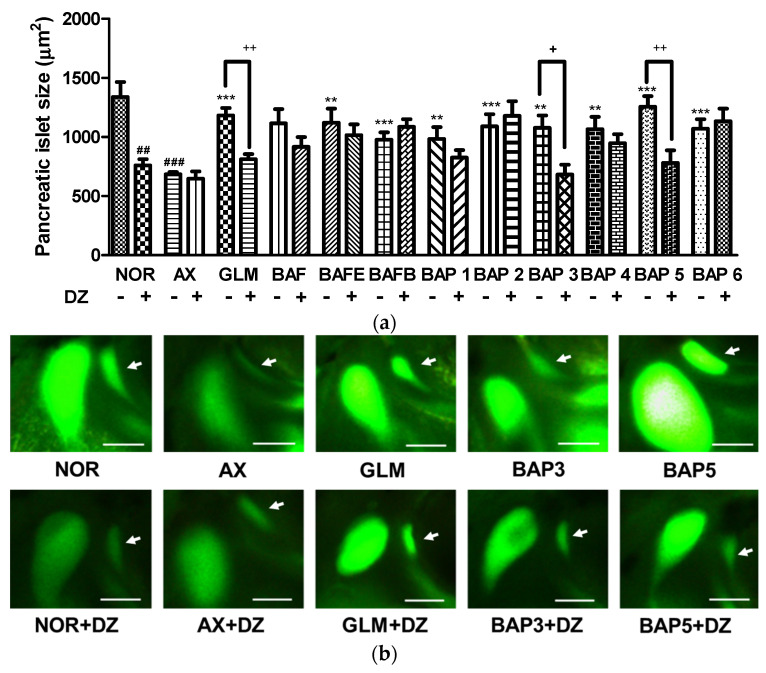
Action of diazoxide (DZ) for pancreatic islets damage by alloxan (AX) in zebrafish fractions and compounds. (**a**) Size of the pancreatic islets. (**b**) Pancreatic islet image. Normal (NOR), NOR+alloxan-treatment (AX), AX+glimepiride, a positive control (GLM), AX+Extract, EtOAc, and *n*-BuOH fractions of *B. arborea* flowers (BAF, BAFE, BAFB), AX + phenylalkyl glucosides **1**–**6** (BAP1~BAP6), treated with and without diazoxide (DZ). (^##^ *p* < 0.01, ^###^ *p* < 0.001; compared to NOR), (** *p* < 0.01, *** *p* < 0.001; compared to AX), (+ *p* < 0.05, ++ *p* < 0.01). Scale bar = 100 μm.

The metabolism of glucose in pancreatic cells is the key step in glucose-stimulated insulin secretion [24]. To study the involvement of the pancreatic *β*-Cell KATP channel stimulation activity, diazoxide (DZ), a K_ATP_ channel opener, was used. The size of the pancreatic islets in the DZ-treated normal group was significantly smaller (43.4%, *p* = 0.0052) relative to the normal group without DZ treatment. Furthermore, the alloxan (AX) group showed no significant difference compared to the DZ-treated group. Pancreatic islet size in the 10 μg/mL glimepiride (GLM), AX, and DZ co-treatment groups was significantly lower (48.9%, *p* = 0.0202) compared to the 10 μg/mL GLM and AX co-treated groups without DZ. Groups co-treated with compounds **1**, **2**, **4**, or **6** and AX were not significantly different after treatment with DZ, indicating no relationship with K_ATP_ channels. Compounds **3** and **5,** in addition to AX, yielded significantly smaller pancreatic islet sizes after treatment with DZ (**3**: 57.6%, *p* = 0.0358; **5**: 69.3%, *p* = 0.0358) compared to the compound+AX groups (Figure 3). These results suggest that compounds **3** and **5** might stimulate insulin secretion by Ca^2+^ influx via the closure of the K_ATP_ channels in *β*-cells.

## 3. Materials and Methods

### 3.1. Plant Materials

The dried flowers of *B. arborea* L. were supplied by Herb Island, Pocheon, Korea, in June 2014 and were identified by Professor Dae-Keun Kim, Woosuk University, Jeonju, Korea. A voucher specimen (KHU2014-0623) is reserved at the Laboratory of Natural Products Chemistry, Kyung Hee University, Yongin, Korea.

### 3.2. General Experimental Procedures

The silica gel (SiO_2_) and octadecyl SiO_2_ (ODS); Kiesel gel 60 (Merck, Darmstadt, Germany), Lichroprep RP-18 (40~60 μm, Merck), and Sephadex LH-20 (Amersham Biosciences, Uppsala, Sweden). Thin layer chromatography (TLC); Kiesel gel 60 F_254_ and RP-18 F_254_S (Merck) TLC plates. The spots were detected using a UV lamp Spectroline Model ENF-240 C/F (Spectronics Corporation, Westbury, NY, USA) and a 10% H_2_SO_4_ solution. Nuclear magnetic resonance (NMR) spectra; 400 MHz FT-NMR spectrometer (Varian Inova AS-400, Palo Alto, CA, USA). Deuterium solvents; Merck Co. Ltd. and Sigma Aldrich Co. Ltd. (St. Louis, MO, USA). IR spectra; Perkin Elmer Spectrum One FT-IR spectrometer (Buckinghamshire, England). Electronic ionization mass spectrometry (EI/MS) and fast atom bombardment mass spectrometry (FABMS) spectra; JEOL JMS-700 (Tokyo, Japan). Melting points; Fisher-John’s melting point apparatus (Fisher Scientific, Miami, FL, USA) without correction.

### 3.3. Extraction and Isolation

*B. arborea* flowers (900 g, in drying) were extracted with 80% aqueous methanol (27 L × 3) at room temperature for 24 h. Concentrated methanol extracts (160 g) were suspended in water (1 L) and then successively partitioned with ethyl acetate (EtOAc, 1 L × 3), *n*-butanol (*n*-BuOH, 0.6 L × 3), and water. The extracts were concentrated to produce the residues of the EtOAc fraction (BAFE, 12 g), the *n*-BuOH fraction (BAFB, 16 g), and the H_2_O fraction (BAFW, 132 g), respectively. The EtOAc fraction was applied to SiO_2_ CC (7.0 × 15.0 cm) and eluted with *n*-hexane-EtOAc (4:1 → 2:1 → 1:1, 2.0 L of each) and CHCl_3_-MeOH-H_2_O (50:3:1 → 36:3:1 → 25:3:1 → 18:3:1 → 12:3:1 → 9:3:1 → 7:3:1 → 6:4:1, 1.5 L of each). The eluting solutions were monitored by TLC to produce 16 fractions (BAFE-1 to BAFE-16). BAFE-10 (966.2 mg, elution volume/total volume (Ve/Vt) 0.649–0.718) was subjected to ODS CC (4.5 × 10 cm) and eluted with acetone-H_2_O (1:2 → 1:1, 3.7 L of both) to yield 16 fractions (BAFE-10-1 to BAFE-10-16). BAFE-10-5 (64.2 mg, Ve/Vt 0.020–0.034) was subjected to SiO_2_ CC (2.5 × 7 cm) and eluted with CHCl_3_-MeOH-H_2_O (30:3:1, 1.2 L) to ultimately produce six fractions (BAFE-10-5-1 to BAFE-10-5-6) along with purified compound **5** [3-phenylpropyl glucoside, BAFE-10-5-4, 13.9 mg, Ve/Vt 0.458–0.733, TLC (SiO_2_ F_254_) R_f_ 0.54, CHCl_3_-MeOH-H_2_O = 9:3:1]. The *n*-BuOH fraction (BAFB) was implemented by SiO_2_ CC (7.0 × 13.0 cm) and eluted with CHCl_3_-MeOH-H_2_O (12:3:1 → 9:3:1 → 6:4:1, 12.0 L of each) yielding 14 parts (BAFB-1 to BAFB-14). BAFB-5 (447.5 mg, Ve/Vt 0.063–0.075) was subjected to Sephadex-LH-20 CC (2.5 × 50 cm) and eluted with MeOH-H_2_O (4:1, 296 mL) to yield six fractions (BAFB-5-1 to BAFB-5-6). BAFB-5-3 (151.9 mg, Ve/Vt 0.446–0.500) was subjected to ODS CC (2.0 × 17 cm) and eluted with MeOH-H_2_O (1:2, 485 mL) yielding nine parts (BAFB-5-3-1 to BAFB-5-3-9) as well as **1 [**benzyl glucoside, BAFB-5-3-3, 88.0 mg, Ve/Vt 0.155–0.196, TLC (SiO2 F_254_) R_f_ 0.44, CHCl_3_-MeOH-H_2_O = 12:3:1] and **3** [2-phenylethyl glucoside, BAFB-5-3-6, 20.5 mg, Ve/Vt 0.289–0.330, TLC (SiO_2_ F_254_) R_f_ 0.44, CHCl_3_-MeOH-H_2_O = 12:3:1]. BAFB-9 (1.2 g, Ve/Vt 0.163–0.225) was subjected to SiO_2_ CC (4.0 × 15.0 cm) and eluted with CHCl_3_-MeOH-H_2_O (10:3:1, 3.1 L) to yield 14 fractions (BAFB-9-1 to BAFB-9-14). BAFB-9-8 (487.7 mg, Ve/Vt 0.308–0.607) was subjected to SiO_2_ CC (3.5 × 15.0 cm) and eluted with EtOAc-*n*-BuOH-H_2_O (22:3:1, 2.7 L) to yield 15 fractions (BAFB-9-8-1 to BAFB-9-8-15). BAFB-9-8-15 (33.7 mg, Ve/Vt 1.000) was implemented by ODS CC (2.0 × 5.0 cm) and eluted with MeOH-H_2_O (1:3, 430 mL) to ultimately produce seven fractions (BAFB-9-8-15-1 to BAFB-9-8-15-7) along with purified **6** [(2*S*, 3*S*)-1-phenyl-2,3-dihydroxybutyl glucosyl-(1→6)-glucoside, BAFB-9-8-15-3, 12.0 mg, Ve/Vt 0.070–0.140, TLC (SiO_2_ F_254_) R_f_ 0.50, EtOAc-*n*-BuOH-H_2_O = 4:5:1]. BAFB-12 (899.3 mg, Ve/Vt 0.392–0.495) was subjected to ODS CC (3.5 × 5.0 cm) and eluted with MeOH-H_2_O (1:3, 2.9 L) to yield 12 fractions (BAFB-12-1 to BAFB-12-12). BAFB-12-5 (182.2 mg, Ve/Vt 0.082–0.149) was subjected to SiO_2_ CC (2.5 × 16.0 cm) and eluted with CHCl_3_-MeOH-H_2_O (9:3:1, 1.1 L) to ultimately produce eight fractions (BAFB-12-5-1 to BAFB-12-5-8) along with purified **4** [2-phenylethyl glucosyl-(1→6)-glucoside, BAFB-12-5-3, 104.3 mg, Ve/Vt 0.407–0.433, TLC (SiO_2_ F_254_) R_f_ 0.54, CHCl_3_-MeOH-H_2_O = 6:4:1]. BAFB-13 (1.8 g, Ve/Vt 0.495–0.575) was subjected to ODS CC (4.0 × 9.0 cm) and eluted with MeOH-H_2_O (1:2, 2.9 L) to yield 13 fractions (BAFB-13-1 to BAFB-13-13). BAFB-13-5 (128.7 mg, Ve/Vt 0.075–0.097) was subjected to ODS CC (2.5 × 7.0 cm) and eluted with MeOH-H_2_O (1:2, 300 mL) to ultimately produce eight parts (BAFB-13-5-1 to BAFB-13-5-8) as well as **2** [benzyl glucosyl-(1→6)-glucoside, BAFB-13-5-2, 10.3 mg, Ve/Vt 0.120–0.140, TLC (SiO_2_ F_254_) R_f_ 0.57, CHCl_3_-MeOH-H_2_O = 6:4:1].

**Benzyl *O*-*β*-D-glucopyranoside** (**1**): White amorphous powder (CH_3_OH); ${\left[\alpha \right]}_{D}^{25}$ −43° (*c* = 0.10, CH_3_OH); positive FABMS *m/z* 293 [M + Na]^+^; IR (KBr, *v*) 3502, 1697, 1530 cm^−1^; ^1^H- and ^13^C-NMR (400 MHz and 100 MHz, CD_3_OD, δ_H_ and δ_C_); Table 2 and Table 3.

**Benzyl *O*-*β*-D-glucopyranosyl-(1→6)-*β*-D-glucopyranoside** (**2**): White amorphous powder (CH_3_OH); ${\left[\alpha \right]}_{D}^{25}$ −90° (*c* = 0.20, CH_3_OH); positive FABMS *m/z* 433 [M + H]^+^; IR(KBr, ν) 3510, 1671, 1600 cm^−1^; ^1^H- and ^13^C-NMR (400 MHz and 100 MHz, CD_3_OD, δ_H_ and δ_C_); Table 2 and Table 3.

**2-phenylethyl *O*-*β*-D-glucopyranoside** (**3**): White amorphous powder (CH_3_OH); ${\left[\alpha \right]}_{D}^{25}$ −35.3° (*c* = 0.50, CH_3_OH); positive FABMS *m/z* 285 [M + H]^+^; IR(KBr, ν) 3368, 1683, 1573 cm^−1^; ^1^H- and ^13^C-NMR (400 MHz and 100 MHz, CD_3_OD, δ_H_ and δ_C_); Table 2 and Table 3.

**2-phenylethyl *O*-*β*-D-glucopyranosyl-(1→6)-*β*-D-glucopyranoside** (**4**): White amorphous powder (CH_3_OH); ${\left[\alpha \right]}_{D}^{25}$ −82° (*c* = 0.20, CH_3_OH); positive FABMS *m/z* 447 [M + H]^+^; IR(KBr, ν) 3370, 1685, 1586 cm^−1^; ^1^H- and ^13^C-NMR (400 MHz and 100 MHz, CD_3_OD, δ_H_ and δ_C_); Table 2 and Table 3.

**3-phenylpropyl *O*-*β*-D-glucopyranoside** (**5**): White amorphous powder (CH_3_OH); ${\left[\alpha \right]}_{D}^{25}$ −29.3° (*c* = 0.75, CH_3_OH); positive FABMS *m/z* 299 [M + H]^+^; IR(KBr, ν) 3364, 1687, 1574 cm^−1^; ^1^H- and ^13^C-NMR (400 MHz and 100 MHz, CD_3_OD, δ_H_ and δ_C_); Table 2 and Table 3.

**(2*S*,3*S*)-1-phenyl-2,3-dihydroxybutyl-*O*-*β*-D-glucopyranosyl-(1→6)-*β*-D-glucopyranoside** (**6**): White amorphous powder (CH_3_OH); ${\left[\alpha \right]}_{D}^{24}$ −23.0° (*c* = 0.10, CH_3_OH); HRFABMS *m/z* 513.1943 [M + Na]^+^ (calcd for C_22_H_34_O_12_Na, 513.1948); IR(KBr, ν) 3352, 1651, 1528 cm^−1^; ^1^H-NMR (600 MHz, CD_3_OD, δ_H_) 7.41 (2H, br. d, *J* = 8.4 Hz, H-2, 6), 7.32 (2H, dd, *J* = 8.4, 8.4 Hz, H-3, 5), 7.26 (1H, m, H-4), 4.30 (1H, d, *J* = 7.8 Hz, H-1″), 4.17 (1H, d, *J* = 7.8 Hz, H-1′), 4.05 (1H, dd, *J* = 12.0, 5.4 Hz, H-6′a), 3.94 (1H, m, H-9), 3.86 (1H, br. d, *J* = 12.0 Hz, H-6″a), 3.76 (1H, dd, *J* = 12.0, 1.8 Hz, H-6′b), 3.72 (1H, overlapped, H-4′), 3.65 (1H, dd, *J* = 12.0, 5.4 Hz, H-6″b), 3.57 (1H, dd, *J* = 7.8, 7.8 Hz, H-4″), 3.42 (1H, m, H-8), 3.35 (2H, overlapped, H-3′, 3″), 3.31 (2H, overlapped, H-5′, 5″), 3.17 (2H, overlapped, H-2′, 2″), 2.93 (1H, dd, *J* = 12.0, 2.4 Hz, H-7a), 2.76 (1H, dd, *J* = 12.0, 6.0 Hz, H-7b), 1.20 (3H, d, *J* = 6.0 Hz, H-10); ^13^C-NMR (150 MHz, CD_3_OD, δ_C_) 140.2 (C-1), 130.0 (C-3, 5), 129.4 (C-2, 6), 127.2 (C-4), 104.8 (C-1″), 104.5 (C-1′), 88.2 (C-9), 78.0 (C-3′), 78.0 (C-3″), 77.8 (C-5′), 77.8 (C-5″), 76.6 (C-8), 75.2 (C-2′), 75.1 (C-2″), 71.9 (C-4′), 71.6 (C-4″), 69.9 (C-6′), 62.8 (C-6″), 39.3 (C-7), 17.4 (C-10); Table 2 and Table 3.

### 3.4. Free Radical Scavenging Activity

The materials, equipment, and methods used for the free radical scavenging assay of extract, solvent fractions, and compounds **1**–**6** from *B. arborea* flowers were described in a previous study [17].

### 3.5. Antidiabetic Activity

#### 3.5.1. Chemicals and Animals

The chemical materials and animal preparation used for the antidiabetic activity are described in a previous study [25].

#### 3.5.2. Ethics Statement

All zebrafish experimental procedures were carried out in accordance with standard zebrafish protocols and were approved by the Animal Care and Use Committee of Kyung Hee University [KHUASP(SE)-15-10].

#### 3.5.3. Evaluation of Recovery Efficacy on Pancreatic Islet Damaged by Alloxan in Zebrafish

The materials, equipment, and methods used for evaluation of recovery efficacy of extract (BAF), solvent fractions (BAFE and BAFB), and compounds **1**–**6** from *B. arborea* flowers on pancreatic islet damaged by alloxan in zebrafish are described in a previous study [25].

#### 3.5.4. Action of Diazoxide on Alloxan-Induced Diabetic Zebrafish

The materials, equipment, and methods used for the action of diazoxide on alloxan-induced diabetic zebrafish are described in a previous study [25].

## 4. Conclusions

This study endeavored to find new active compounds of *B. arborea* flowers. Six phenylalkyl glucosides, including one new phenylbutyl diglucoside, were isolated through repeated column chromatography using SiO_2_, ODS, and Sephadex LH-20 resins and identified by the analysis of NMR, IR, UV, and FABMS data. Extracts, solvent fractions, and some compounds from *B. arborea* flowers were found to show scavenging activity in ABTS radicals with levels of significance. The EtOAc fraction (BAFE) showed the highest scavenging capacity, whereas compounds **2**, **4**, and **6** did not display a capacity to use the DPPH radical. Compounds **2**, **4**, and **6** were isolated from the BuOH fraction (BAFB). Therefore, the low antioxidant capacity of BAFB compared to the BAFE is expected to be due to the lower antioxidant capacity of these compounds. And the significant antioxidant activity of compound 5 is expected to be one of the reasons for the high antioxidant activity of the BAFE. All compounds and solvent fractions were also found to show protective activity against alloxan-induced pancreatic islet damage in zebrafish larvae with high levels of significance. In particular, compounds **3** and **5** stimulated insulin secretion by Ca^2+^ influx via closure of K_ATP_ channels in *β*-cells. These results indicate that *B. arborea* flowers and their isolated compounds are used as potential antioxidant and antidiabetic agents.

## Figures and Tables

**Table 1:** Radical scavenging capacity of extract, solvent fractions, and isolated compounds from *Brugmansia arborea* L. flowers using ABTS and DPPH radicals.

Samples	Antioxidant Capacity (mg VCE ^1^·g^−1^ DW ^2^)	
	ABTS	DPPH
Compound **1**	50.7 ± 1.5 ^d3^	5.2 ± 0.5 ^c^
Compound **2**	19.9 ± 1.9 ^f4^	N.D. ^8^
Compound **3**	27.4 ± 0.7 ^e^	3.1 ± 0.5 ^d^
Compound **4**	8.6 ± 2.2 ^g^	N.D.
Compound **5**	15.6 ± 2.5 ^f^	2.6 ± 1.1 ^d^
Compound **6**	3.1 ± 0.4 ^h^	N.D.
BAF ^5^	64.3 ± 1.3 ^b^	16.2 ± 1.8 ^b^
BAFB ^6^	56.4 ± 0.2 ^c^	20.1 ± 0.2 ^b^
BAFE ^7^	513.2 ± 12.9 ^a^	130.9 ± 3.7 ^a^

^1^ VCE stands for vitamin C equivalent. ^2^ DW stands for dry weight. ^3^ Data are presented as the mean ± standard deviations (*n* = 3). ^4^ Means with different superscripts in the same column are significantly different by Tukey–Kramer’s HSD (*p* < 0.05). ^5^ Extract from *Brugmansia arborea* L. flowers. ^6^ *n*-BuOH fraction from *Brugmansia arborea* L. flowers. ^7^ EtOAc fraction from *Brugmansia arborea* L. flowers. ^8^ No determined.

**Table 2:** ^1^H-NMR spectral data (CD_3_OD; 400 MHz) of compounds **1**–**6**. δ in ppm, *J* in Hz.

No.	δ_H_, *J* in Hz					
	1	2	3	4	5	6
2	7.41, br. d, 8.4	7.41, br. d, 8.4	7.41, br. d, 8.4	7.41, br. d, 8.4	7.41, br. d, 8.4	7.41, br. d, 8.4
3	7.32, dd, 8.4, 8.4	7.32, dd, 8.4, 8.4	7.32, dd, 8.4, 8.4	7.32, dd, 8.4, 8.4	7.32, dd, 8.4, 8.4	7.32, dd, 8.4, 8.4
4	7.26, m	7.26, m	7.26, m	7.26, m	7.26, m	7.26, m
5	7.32, dd, 8.4, 8.4	7.32, dd, 8.4, 8.4	7.32, dd, 8.4, 8.4	7.32, dd, 8.4, 8.4	7.32, dd, 8.4, 8.4	7.32, dd, 8.4, 8.4
6	7.41, br. d, 8.4	7.41, br. d, 8.4	7.41, br. d, 8.4	7.41, br. d, 8.4	7.41, br. d, 8.4	7.41, br. d, 8.4
7	4.92, d, 11.6 4.66, d, 11.6	4.92, d, 11.6 4.66, d, 11.6	2.92, t, 7.2	2.93, t, 7.2	2.80, t, 7.2	2.93, dd, 12.0, 2.4 2.76, dd, 12.0, 6.0
8	-	-	4.05, m 3.74, m	4.07, m 3.76, m	2.92, m	3.42, m
9	-	-	-	-	4.05, m 3.74, m	3.94, m
10	-	-	-	-	-	1.20, d, 6.0 (3H)
1′	4.34, d, 7.6	4.34, d, 8.0	4.30, d, 8.0	4.36, d, 8.0	4.30, d, 8.0	4.17, d, 7.8
2′	3.23 ^a^)	3.23 ^a^)	3.17 ^a^)	3.23 ^a^)	3.17 ^a^)	3.17 ^a^)
3′	3.35 ^a^)	3.35 ^a^)	3.35 ^a^)	3.35 ^a^)	3.35 ^a^)	3.35 ^a^)
4′	3.27 ^a^)	3.27 ^a^)	3.27 ^a^)	3.27 ^a^)	3.27 ^a^)	3.72 ^a^)
5′	3.31 ^a^)	3.31 ^a^)	3.31 ^a^)	3.31 ^a^)	3.31 ^a^)	3.31 ^a^)
6′	3.88, dd, 12.0, 2.0 3.66, dd, 12.0, 5.6	3.88, dd, 12.0, 2.0 3.66, dd, 12.0, 5.6	3.86, dd, 12.0, 2.0 3.65, dd, 12.0, 5.6	3.85, dd, 12.0, 2.0 3.65, dd, 12.0, 5.6	3.86, dd, 12.0, 2.0 3.65, dd, 12.0, 5.6	4.05, dd, 12.0, 5.4 3.76, dd, 12.0, 1.8
1″	-	4.90 ^a^)	-	4.34, d, 8.0	-	4.30, d, 7.8
2″	-	3.38 ^a^)	-	3.38 ^a^)	-	3.17 ^a^)
3″	-	3.52 ^a^)	-	3.52 ^a^)	-	3.35 ^a^)
4″	-	3.45 ^a^)	-	3.45 ^a^)	-	3.57, dd, 7.8, 7.8
5″	-	3.48 ^a^)	-	3.48 ^a^)	-	3.31 ^a^)
6″	-	4.67, br. d, 12.0 4.39, dd, 12.0, 6.0	-	4.25, dd, 11.6, 2.0 3.75, dd, 11.6, 6.0	-	3.86, br. d, 12.0 3.65, dd, 12.0, 5.4

^a^) Overlapped signals, reported without designated multiplicity.

**Table 3:** ^13^C-NMR spectral data (CD_3_OD; 100 MHz) of compounds **1**–**6**. δ in ppm.

No.	δ_C_					
	1	2	3	4	5	6
1	139.0	139.0	139.0	139.0	139.0	140.2
2	129.1	129.1	129.1	129.1	129.1	129.4
3	129.2	129.2	129.2	129.2	129.2	130.0
4	128.6	128.6	128.6	128.6	128.6	127.2
5	129.2	129.2	129.2	129.2	129.2	130.0
6	129.1	129.1	129.1	129.1	129.1	129.4
7	71.7	71.5	37.2	37.2	40.2	39.3
8	-		71.7	71.5	37.2	76.6
9	-		-	-	71.7	88.2
10	-	-	-	-	-	17.4
1′	103.2	103.4	103.4	103.4	103.4	104.5
2′	75.1	75.0	75.0	75.0	75.0	75.2
3′	78.0	78.0	78.0	78.0	78.0	78.0
4′	71.6	71.5	71.6	71.5	71.6	71.9
5′	78.0	77.9	77.9	77.9	77.9	77.8
6′	62.8	69.7	62.7	69.7	62.7	69.9
1″	-	104.8	-	104.8		104.8
2″	-	75.1	-	75.1		75.1
3″	-	77.8	-	77.8		78.0
4″	-	71.4	-	71.4		71.6
5″	-	77.1	-	77.1		77.8
6″	-	62.7	-	62.7		62.8

## Data Availability

The ^1^H, ^13^C, gHMBC, gHSQC, HRFABMS, and IR spectra of compound **6** are available from the supplementary materials.

## Supplementary Materials

The following supporting information can be downloaded at: https://www.mdpi.com/article/10.3390/plants12244075/s1, Figure S1: ^1^H NMR spectrum of brugmansioside C (**6**) in CD_3_OD (600 MHz); Figure S2: ^13^C NMR spectrum of brugmansioside C (**6**) in CD_3_OD (150 MHz); Figure S3: gHMBC spectrum of brugmansioside C (**6**) in CD_3_OD; Figure S4: gHSQC spectrum of brugmansioside C (**6**) in CD_3_OD; Figure S5: HRFABMS spectrum of brugmansioside C (**6**); Figure S6: IR spectrum of brugmansioside C (**6**).
