# Supplementary material for: Phenylalkyl Glycosides from the Flowers of Brugmansia arborea L. and Their Radical Scavenging Effect and Protective Effect on Pancreatic Islets Damaged by Alloxan in Zebrafish (Danio rerio) Larvae
Source: Plants (Basel). 2023 Dec 5;12(24):4075. doi: 10.3390/plants12244075 (PMC10747368; doi:10.3390/plants12244075)

SUPPLEMENTARY MATERIAL

Phenylalkyl glycosides from the flowers of *Brugmansia arborea* L. and their radical scavenging effect and protective effect on pancreatic islets damaged by alloxan in zebrafish (*Danio rerio*) larvae

Hyoung-Geun Kim[1], Youn Hee Nam[1], Min-Ho Lee[2], Tong Ho Kang[1], Nam-In Baek[1], and Daeyoung Lee[3*]

[1] Graduate School of Biotechnology and Department of Oriental Medicinal Biotechnology, Kyung Hee University, Yongin 17104, Republic of Korea

[2] Department of Food Technology and Services, Eulji University, Seongnam 13135, Republic of Korea

[3] Department of Herbal Crop Research, National Institute of Horticultural and Herbal Science, RDA, Eumseong 27709, Republic of Korea

Contents

Figure S1. ^{1}H NMR spectrum of brugmansioside C (**6**) in CD_3OD (600 MHz)

Figure S2. ^{13}C NMR spectrum of brugmansioside C (**6**) in CD_3OD (150 MHz)

Figure S3. gHMBC spectrum of brugmansioside C (**6**) in CD_3OD

Figure S4. gHSQC spectrum of brugmansioside C (**6**) in CD_3OD

Figure S5. HRFABMS spectrum of brugmansioside C (**6**)

Figure S6. IR spectrum of brugmansioside C (**6**)

Figure S1. ^{1}H NMR spectrum of brugmansioside C (**6**) in CD_3OD (600 MHz)

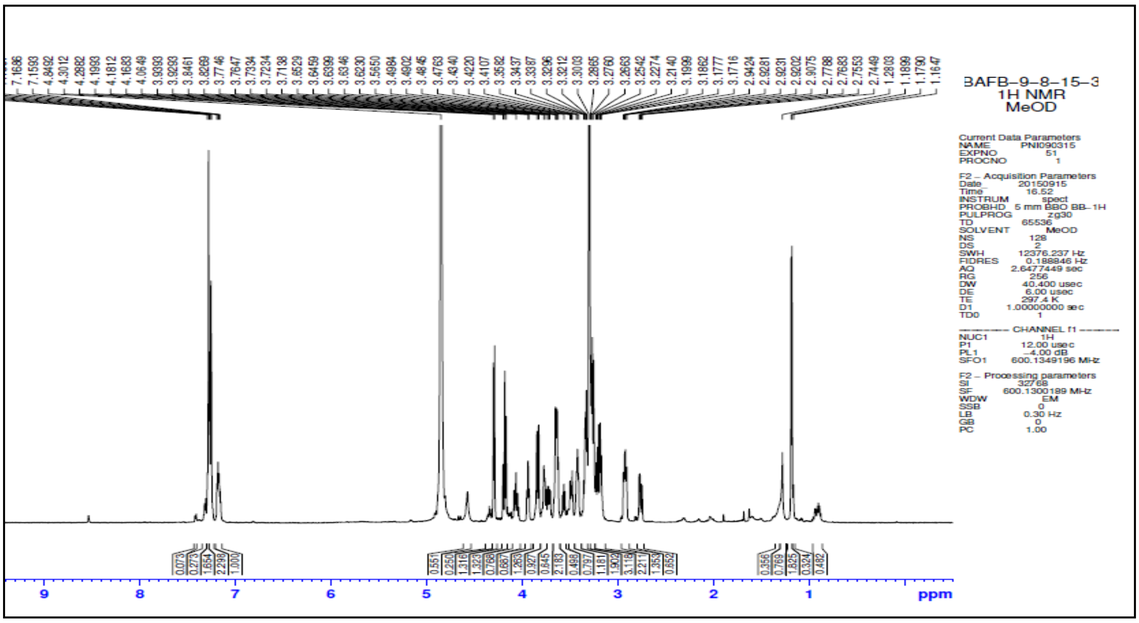

Figure S2. ^{13}C NMR spectrum of brugmansioside C (**6**) in CD_3OD (150 MHz)

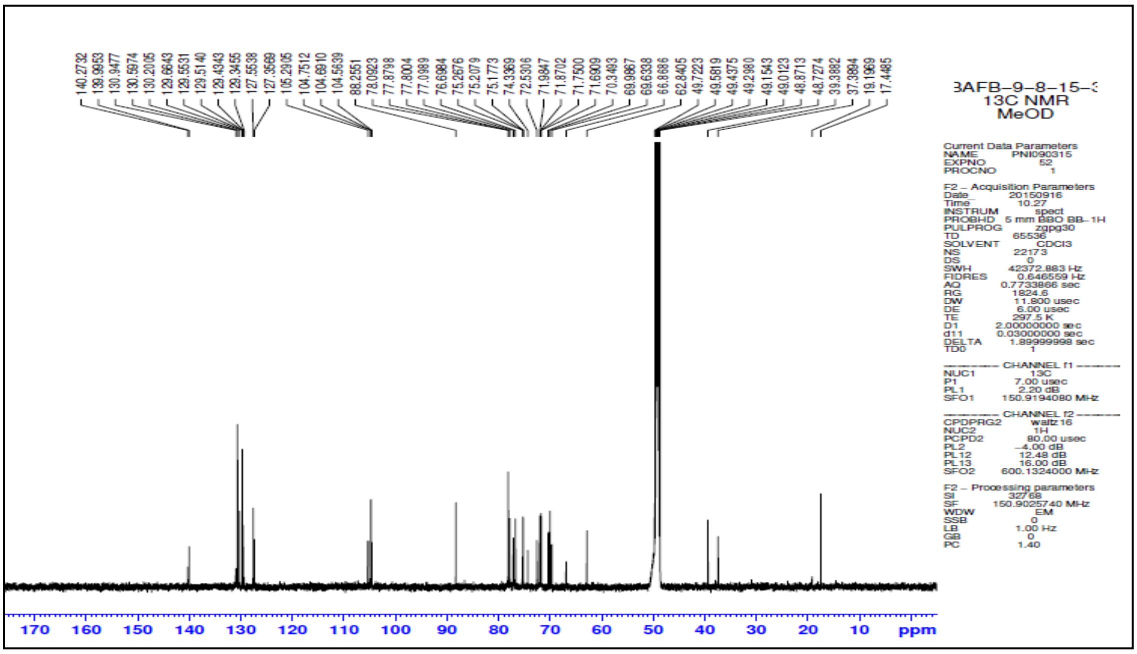

Figure S3. gHMBC spectrum of brugmansioside C (**6**) in CD_3OD (600 MHz)

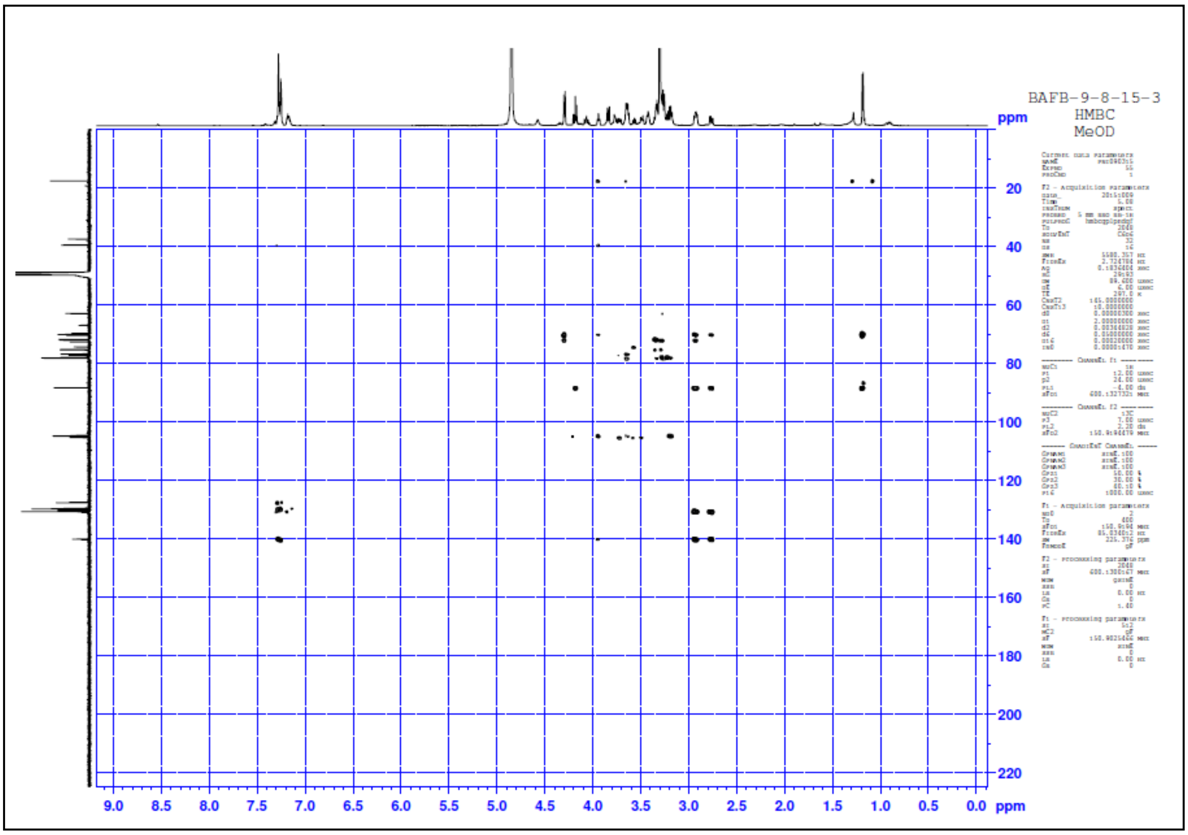

Figure S4. gHSQC spectrum of brugmansioside C (**6**) in CD_3OD (600 MHz)

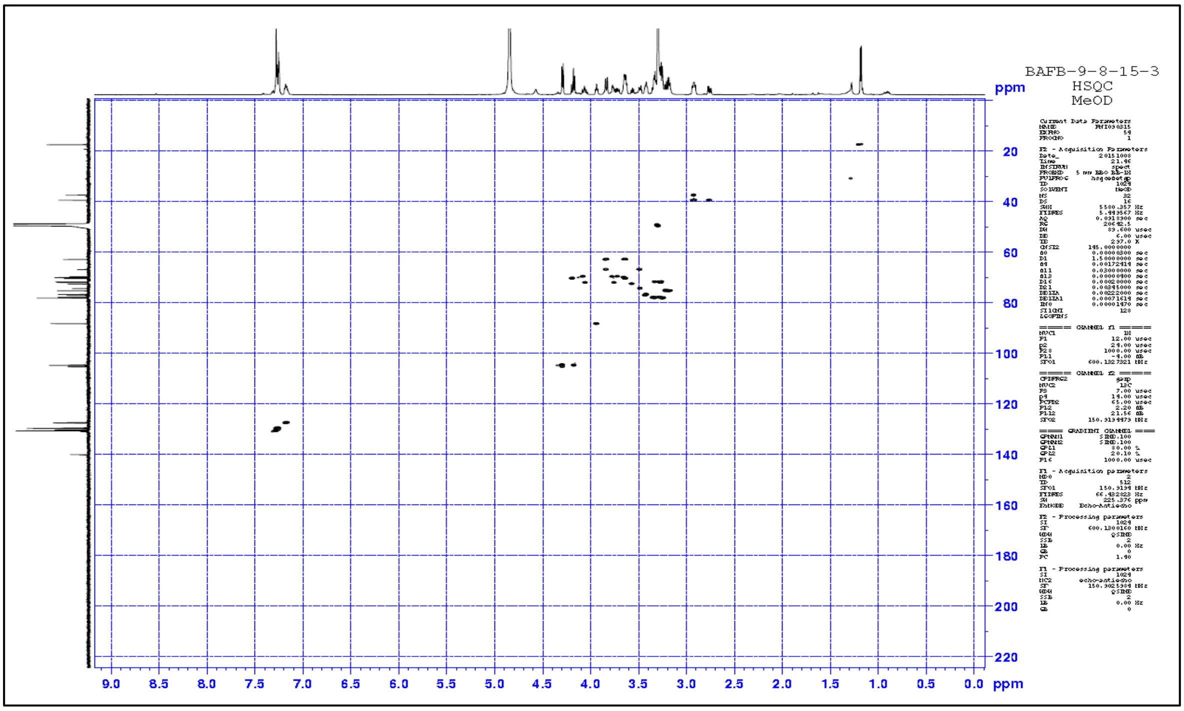

Figure S5. HRFABMS spectrum of brugmansioside C (**6**)

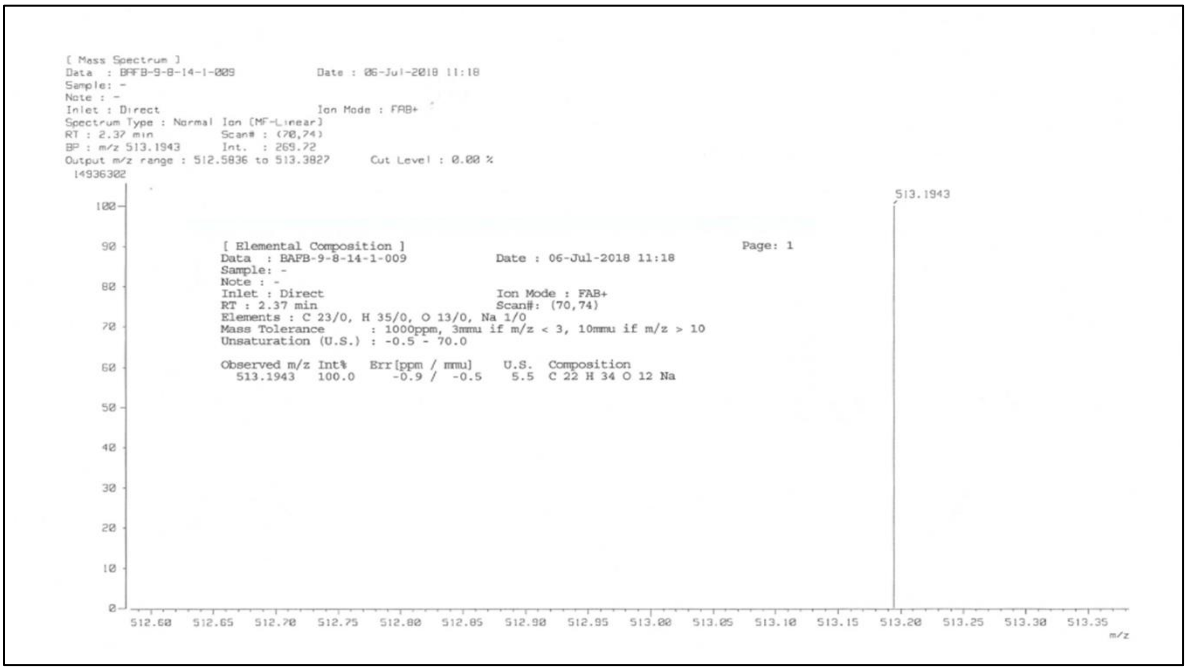

Figure S6. IR spectrum of brugmansioside C (**6**)

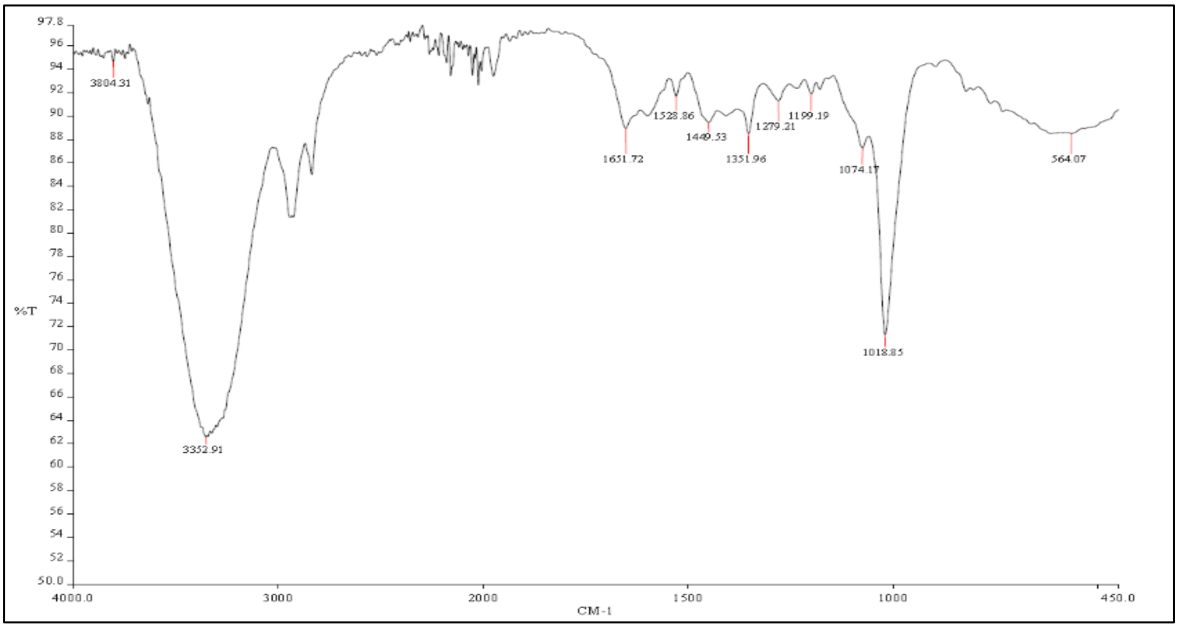

Supplement: Supplementary file 1 [file plants-12-04075-s001.zip › plants-2735982-supplementary.pdf]
